# Theatre Is a Valid Add-On Therapeutic Intervention for Emotional Rehabilitation of Parkinson's Disease Patients

**DOI:** 10.1155/2017/7436725

**Published:** 2017-11-22

**Authors:** Giovanni Mirabella, Paolo De Vita, Michele Fragola, Silvia Rampelli, Francesco Lena, Fulvia Dilettuso, Marta Iacopini, Raffaella d'Avella, Maria Concetta Borgese, Silvia Mazzotta, Deborah Lanni, Marco Grano, Sara Lubrani, Nicola Modugno

**Affiliations:** ^1^Istituto Neurologico Mediterraneo “Neuromed”, Pozzilli, Italy; ^2^Department of Anatomy, Histology, Forensic Medicine & Orthopedics, Sapienza University of Rome, Rome, Italy; ^3^PARKIN-ZONE onlus, Roma, Italy

## Abstract

Conventional medical treatments of Parkinson's disease (PD) are effective on motor disturbances but may have little impact on nonmotor symptoms, especially psychiatric ones. Thus, even when motor symptomatology improves, patients might experience deterioration in their quality of life. We have shown that 3 years of active theatre is a valid complementary intervention for PD as it significantly improves the well-being of patients in comparison to patients undergoing conventional physiotherapy. Our aim was to replicate these findings while improving the efficacy of the treatment. We ran a single-blinded pilot study lasting 15 months on 24 subjects with moderate idiopathic PD. 12 were assigned to a theatre program in which patients underwent “emotional” training. The other 12 underwent group physiotherapy. Patients were evaluated at the beginning and at the end of their treatments, using a battery of eight clinical and five neuropsychological scales. We found that the emotional theatre training improved the emotional well-being of patients, whereas physiotherapy did not. Interestingly, neither of the groups showed improvements in either motor symptoms or cognitive abilities tested by the neuropsychological battery. We confirmed that theatre therapy might be helpful in improving emotional well-being in PD.

## 1. Introduction

Parkinson's disease is a progressive neurodegenerative disease that causes motor disturbances (e.g., slowness, rigidity, tremor, and disorders of gait and balance) and nonmotor disturbances such as neuropsychiatric symptoms (e.g., depression, anxiety, obsessive-compulsive disorders, and cognitive impairments) and autonomic dysfunction (e.g., decreased control of urinary bladder and sexual dysfunctions) [1]. Consequently, the general health and the social lives of patients can be deeply impaired [2].

Standard medical treatments based on the administration of dopaminergic drugs [3, 4] allow optimal control of the motor symptoms, especially in the initial stages of PD; however, with chronic treatment, motor and behavioral complications partly unmasked by dopaminergic medications may develop [5]. Moreover, dopaminergic drugs are not often fully effective in controlling the full clinical spectrum of nonmotor symptoms. Often physiotherapy is used as an additional therapy [6], but even though it has been shown to be effective [7, 8], benefits tend to disappear as soon as the treatment is over [9] and there is no clear evidence of its efficacy on nonmotor symptoms.

Thus, as PD progresses, the increasing difficulties in its management may lead to social isolation as patients start to feel embarrassed by the disease, with a consequent deterioration in their quality of life (QoL; [10]). That said, there is a gap between the effects of the best available medical treatment and patients' expectations. In some instances, a paradoxical discrepancy between an objectively good control of motor disturbances and an increasing negative feeling of well-being reported by patients may occur.

Given that PD has worldwide prevalence of approximately 10 million people and that this number is expected to double by 2050 because of increasing longevity [11], management and treatment of this disease will be a very important public health problem. Accordingly, the demand for the development of appropriate complementary strategies aimed at improving personal and social life of patients and caregivers is rapidly increasing. Activities such as tai chi [12], dancing tango [13], and Irish dance [14] have been shown to produce positive effects mainly on balance and frequency of falls. However, improvements in nonmotor cognitive and affective symptoms were absent or very limited. An exception is given in a study [15] that demonstrated that 3 months of active music therapy improved both motor abilities and the emotional status of PD patients. Nevertheless, these effects disappeared 2 months after the end of therapy. Some preliminary evidence indicates that group psychotherapy [16] and occupational therapies [17, 18] might be a useful treatment for emotional disorders such as depression and/or anxiety, but samples are usually very small, measurements are too few, and control groups are not always present. Therefore, more systematic research is necessary to quantify the effects of those approaches.

Recently, we have shown that active theatre, in which patients are directly involved in the representations, is a valid add-on therapeutic intervention for PD [19]. Compared to patients undergoing physiotherapy, only PD patients performing theatre had progressive improvements in most nonmotor clinical scales (especially those tapping into the affective domains) and, to a lesser extent, in those assessing motor symptoms. In particular, patients performing theatre showed remarkable improvements in their level of depression, in their self-esteem, and in the quality of sleep. However, most of these beneficial effects emerged only after a training of 3 years and this evidence casts doubts on the transferability of the theatre therapy program. In addition, our sample was relatively small (20 subjects). Finally, as only five clinical scales were used, they might not be enough to fully appreciate the effects of the interventions. The present study was designed to both (i) replicate and extend our previous results, by collecting data on more clinical and neuropsychological scales, and (ii) develop a form of theatre therapy which would speed up the emergence of benefits in order to improve its transferability. Because in the previous study PD patients showed a significant improvement in most of clinical scales evaluating the emotional sphere, we hypothesized that a way to make theatre training more efficient would have been to train patients to represent emotional events on the stage. The effects of such “emotional” theatre were compared with those induced by physiotherapy in other groups of PD patients.

## 2. Materials and Methods

### 2.1. Study Participants

Forty-five patients were recruited from the outpatients of several hospitals in Rome (*n* = 25) and from IRCCS Neuromed Hospital, Italy (*n* = 20), by means of referrals from neurologists and of advertising through local PD associations. Eligibility criteria for including PD patients were (i) a diagnosis of idiopathic PD with a moderate disease severity (Hoehn-Yahr stage 2-3), (ii) a stable treatment with levodopa (L-dopa) and dopamine agonists, (iii) absence of cognitive impairment (MMSE score ≥ 24), (iv) absence of severe sensory deficits, (v) absence of severe motor disability so that they could stand and walk unaided, and (vi) not being involved in other rehabilitation studies.

We allowed patients recruited from Rome hospitals to be assigned to the theatre rehabilitation program* (theatre group)*, while patients from Neuromed Hospital were allowed to enter in the physiotherapy rehabilitation therapy* (control group)*. Two main motivations produced this choice: (1) actors were only available in Rome; (2) the two groups of patients would not be in contact (Neuromed Hospital is about 200 km from Rome), avoiding possible complaints about being assigned to a given group.

After the initial screening, patients who met inclusion criteria underwent a one-to-one interview (sometimes by telephone) led by one member of the staff who explained the type of study in which they were taking part. Ten patients refused to participate in the theatre group and five in the physiotherapy group. Motivations were mainly related to the programmed length of the study or to logistic problems. Of the remaining 30 patients, six (three from each group) did not conclude the study because (a) two moved to another town; (b) three experienced physical problems; and (c) one had lack of motivation (1). We ended up having 24 patients, 12 per group, who took part in more than 75% of the study sessions. Throughout the entire course of the study, patients were allowed to continue taking their dopaminergic therapy, which was optimized whenever necessary according to the patient's needs. The use of antidepressant and hypnotic agents was equally distributed between the two groups during the entire course of the study.

All subjects gave their informed consent and were free to withdraw from the study at any time. The procedures were approved by the local Institutional Ethics Committee and were in accordance with the ethical standards laid down in the Declaration of Helsinki of 1964.

### 2.2. Clinical Assessment

All patients underwent a clinical evaluation at the beginning (*T*0) of the training period and after 15 months (*T*1). A neurologist and a psychologist, blinded to the study groups, evaluated PD patients on clinical and neuropsychological scales, respectively. We employed eight clinical scales: (i) the Unified Parkinson's Disease Rating Scale (UPDRS), which rates patients' mood and cognition (UPDRS I), activities of daily living (UPDRS II), motor symptoms (UPDRS III), and complications of therapy (UPDRS IV); (ii) the Gait and Falls Questionnaire, which measures gait disturbances; (iii) the Parkinson's Disease Quality of Life Scale (PDQ39), which measures the QoL by summing the scores of its eight subscales (mobility, activities of daily living, emotional well-being, stigma, social support, cognition, communication, and bodily discomfort); (iv) the Beck Depression Inventory, which measures the level of depression; (v) the Apathy Evaluation Scale, which measures the level of apathy (lack of feelings, emotions, interests, or concerns); (vi) the Hamilton Anxiety Rating Scale, which measures the level of anxiety; (vii) the Parkinson's Disease Sleep Scale (PDSS), which measures sleep and nocturnal disability in PD; and (viii) the Schwab and England Scale, which assesses the degree of functional independence in daily living. In addition, we assessed some cognitive functions exploiting five neuropsychological tests: (i) the Raven test, which measures general intelligence; (ii) the Stroop test, which measures attention and/or inhibitory functions; (iii) the Rey test, which measures verbal memory; (iv) the digit span task, which measures working memory's capacity for numbers; and (v) the phonological fluency test, which measures the ability of participants to generate words that begin with a given letter.

The rating of all scales was carried out in their best ON medication state calculated after the first morning dose which normally allowed the patient to attain the best control of symptoms. As a result, patients were rated 45–60 minutes after the administration of an L-dopa dose ranging from 100 to 200 mg.

### 2.3. Emotional Theatre Workshop

The theatrical workshop consisted of 3-hour daily session, once a week, giving a total of ~12 h/month for 15 months. Each session was led by two professionals, an actor and in turn a dancer or a singer. Approximately 50 minutes were spent performing either movement or voice training, while the following 50 minutes were spent in theatrical training (see Table 1 and Movies 1 and 2 in Supplementary Material available online at https://doi.org/10.1155/2017/7436725). All exercises were organized according to a theme (e.g., the experience and expression of anger). All the remaining time (~80 minutes) was focused on performing theatre scenes or theatrical tasks that required the representation of the chosen theme. The whole program was divided into three phases: (i) welcome, self-confidence, and group foundation (~4 months); (ii) emotional stress work focusing on six different emotional moods: anger, fear, happiness, sadness, surprise, and sensuality (~8 months); and (iii) free organization, interpretation, and representation of emotional states by each patient using either texts or improvisations and/or body movements (~3 months).

### 2.4. Physiotherapy

Physiotherapy consisted of 1.5-hour group sessions, 2 days a week, giving a total of ~12 h/month for 15 months. Each session was led by a physiotherapist. The physical therapy program was designed to increase strength, power, endurance, and aerobic capacity and to improve motor functions, postural control, balance, and gait according to the European Physiotherapy Guideline for Parkinson's Disease (for more details, see Table 2).

### 2.5. Statistics

Using the Shapiro-Wilk test, we verified that, in the vast majority of instances, the assumption of normality was verified (108/112 or 96.4%; see Table 3), with the exception of data recorded with the Schwab and England Scale. Thus, in the latter case, we used the Wilcoxon signed-rank test for comparisons within a group and the Mann–Whitney* U* test for comparisons between groups, lowering the alpha value according to the number of comparisons (alpha: 0.05/4 = 0.0125). For all the other scales, a two-way mixed-design ANOVA [between-subjects factor: GROUP (theatre; controls); within-subjects factor: TIME (*T*0, *T*1)] was employed for assessing changes in the scores across the experimental conditions. Bonferroni corrections were applied to all post hoc tests (pairwise comparisons). In order to provide a measure of the “effect size,” we computed the partial eta-squared (*η*_*p*_^2^) for each ANOVA, with values of 0.139, 0.058, and 0.01 indicating large, medium, and small effects, respectively, and Cohen's* d* as the effect size for* t*-tests, with values of 0.2, 0.5, and 0.8 indicating large, medium, and small effects [20]. As *η*_*p*_^2^ and Cohen's* d* allow comparison between the effects of a given manipulation regardless of other factors that have been manipulated, they allow comparison of our results with those of future studies.

## 3. Results

As shown in Table 4, at time *T*0, demographical and clinical data did not differ between the two groups. The levels of instruction were similar, preventing a possible criticism that people from a great metropolis, such as Rome, may be more educated than those coming from more rural areas.

A two-way mixed-design ANOVA on the amount of L-dopa equivalent daily dose (LEDD) (mg) administered did not show a main effect of group (*M*_diff_ = 117; SD = 123) (*F*(1,22) = 0.91;* p* = 0.35; *η*_*p*_^2^ = 0.04; 95% CI [−137; 373]) or a main effect of time (*M*_diff_ = 15; SD = 39) (*F*(1,22) = 0.15;* p* = 0.69; *η*_*p*_^2^ = 0.07; 95% CI [−65; 95]) or an interaction (*F*(1,22) = 2.22; *p* = 0.15; *η*_*p*_^2^ = 0.09). However, theatre-group patients on average had a small decrease in dopaminergic drug therapy during the course of the study (LEDD = 34.1 mg), while control patients needed on average an increase in dopaminergic drug therapy (LEDD = 73.8 mg).

**Figure 1:**
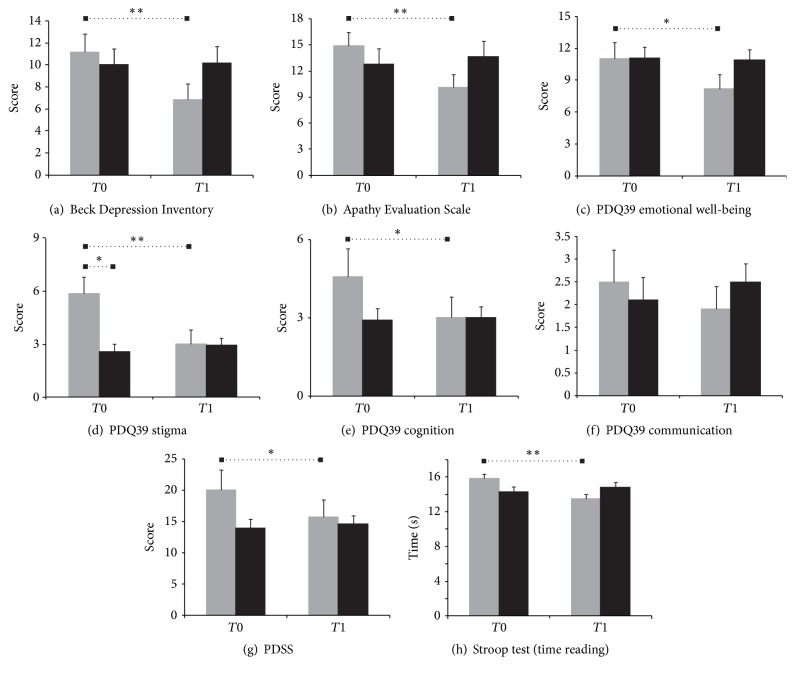
Mean scores and standard error of the means at the two time points for the theatre (gray bars) and control group (black bars) in the scales or subscales showing a significant effect at the mixed-design ANOVAs (see text and Table 5 for more details). Bars indicate significant differences after post hoc analyses, the single *∗* indicates values of *p* < 0.01, and the double *∗* indicates values of *p* < 0.001. PDQ39: Parkinson's Disease Quality of Life Scale; PDSS: Parkinson's Disease Sleep Scale.

All the other effects of theatrical training are reported in Tables 5, 6, and 7. None of the clinical scales measuring motor symptoms (UPDRS III, GFQ, and PDQ39-mobility), daily activities (UPDRS II, Schwab and England Scale (The Wilcoxon signed-rank test did not show any significant difference between *T*0 and *T*1 either in theatre patients (*p* = 0.65) or in controls (*p* = 1). The Mann–Whitney* U* test did not show differences between theatre patients and controls either at *T*0 (*p* = 0.89) or at *T*1 (*p* = 0.55).), and PDQ39-activities of daily living), or physical problems (UPDRS IV and PDQ39-bodily discomfort) showed significant differences either within or between the two groups. The same applied to cognitive functions measured either by the UPDRS I or by the neuropsychological tests, with the exception of the reading time of the Stroop test. Here, the main effect of time indicates that, overall, patients increased the speed of reading over the 15 months. This effect was qualified by the interaction group*∗*time, which showed that only theatre patients improved their reading time (Tables 5, 6, and 7 and Figure 1).

While motor symptoms and cognitive functions were not affected by either treatment, four scales addressing the emotional/affective domains, that is, those measuring depression, apathy, stigma, and emotional well-being, showed significant improvements from *T*0 to *T*1 in the theatre group but not in the control group (Tables 5, 6, and 7 and Figure 1). In all these cases, the main effect of the factor time was explained by the interaction group*∗*time, which showed a significant improvement in theatre patients and no changes in the controls over the period of treatment. The levels of anxiety and the social support (PDQ39-social support) had a very similar tendency, but the decrease in anxiety in the theatre group did not reach significance. In addition, the theatre training led to an improvement in the quality of sleep (PDSS), the cognition measured by the subscale of the PDQ39 and, more marginally, the ability to communicate (PDQ39-communication).

## 4. Discussion

The present study successfully replicates our previous results by confirming the efficacy of theatre therapy as an add-on rehabilitative tool for PD patients [19]. The novel finding is that theatre training based on the representation of emotions speeds up the appearance of benefits in the affective domain. We will discuss why theatre might represent a very effective form of cognitive rehabilitation.

### 4.1. Why Is Theatre Training Effective?

Even though motor disturbances are mandatory to make the diagnosis of PD, there is now a consensus around the idea that this is not a pure motor disorder but a multifaceted one. Several factors contribute to its severity. Nonmotor symptoms have been shown to play a more important role than motor symptoms in reducing the health-related and perceived QoL in PD [21, 22]. In particular, depression seems to deeply affect the feelings about the disease perception and its future consequences [21, 23].

Recent findings have started to shed light on the link between the depletion of dopaminergic neurons occurring in PD and the genesis of neuropsychiatric nonmotor symptoms. The disruption of the dopaminergic system seems to affect the decision-making processes underlying the genesis of motor acts (see [24]) because of a wrong evaluation of the costs of movements [25, 26], making this cost too high [26]. The wrong value assignment decreases the motivation to act, inducing either bradykinesia or akinesia and even apathy. Other evidence indicates that, in healthy subjects, L-dopa not only plays a role during decision-making under risky choices but also has a critical role in generating a subjective feeling of happiness which follows the receipt of reward [27]. Therefore, the loss of dopamine is likely to affect both the planning of actions and the pleasant feelings normally associated with rewarding events possibly leading to anhedonia, a core symptom of major depression. Overall, it is not surprising that an objectively good control of motor symptoms might not be coupled with a positive feeling of well-being experienced by patients. This evidence has prompted the need for developing auxiliary approaches to medical therapy. However, the effectiveness of these approaches had rarely been explored with proper randomized controlled trials (for a review, see [28, 29]). Most studies suffer from having small and/or heterogeneous samples, and sometimes methodologies are not rigorous enough (see [12] for a remarkable exception). In addition, the effects of most interventions do not last more than a few months, a span of time that is very likely too short to promote the so-called brain plasticity, that is, the neural mechanism underlying behavioral changes (e.g., [30, 31]). Moreover, another factor has to be considered to evaluate the effectiveness of a therapy, that is, the different degrees to which it affects motor, emotional, and interpersonal components. For instance, art therapies based on drawing, painting, sculpting, or music playing allow patients to express themselves spontaneously, but they tend to lack the intersubjective interactions normally occurring in real life, and their impact on body motor control is rather limited. Dance and martial arts require physical involvement but they do not reproduce the intersubjective interactions of real life. The opposite holds true for occupational therapies. In contrast, theatre training allows a more holistic approach as, to successfully impersonate a character, an actor needs to control his body, reproduce the character's emotions and way of thinking, and identify himself with his social role. To some extent, an actor needs to learn a way to become another person on the stage and to behave accordingly. This is potentially a successful exercise through which PD patients could develop new strategies for carefully controlling their bodies and minds within a protected environment, that is, in a place where they do not feel judged by others. In addition, both during and outside the performance, patients have to continuously interact and thus they are forced to socialize. These unique features make theatre an ideal playground for deeply motivating patients, allowing them to reacquire the control of their social, psychological, and emotional life and to transfer these abilities to everyday life situations.

### 4.2. The Effectiveness of Emotional Theatre

A novel finding of the current work is that the emotional theatre training boosts the improvements in the emotional sphere, allowing the emergence of significant ameliorations in more clinical scales tapping the affective domain than in our previous study [19]. After 15 months, there were no improvements in any of the clinical scales measuring either motor symptoms, daily activities, or physical problems. Nevertheless, even though patients still suffered from physical discomforts, their feelings about the disease and about its evolution were so positive that they declared they could not stop their theatrical activities (Movies 3 and 4). The improvements in the quality of sleep are likely to be associated with the increased psychological well-being. This is in keeping with the idea that improvements in mood bring about an improved perception of the disease [21, 22, 32]. It must be remarked that performing this type of theatre training is not an enjoyable activity by definition. Patients experienced some form of distress when playing emotionally negative events (e.g., the feeling of being powerless in front of someone else; see Movie 2), and some of them reexperienced, directly or indirectly, painful situations (e.g., the loss of some beloved person). Therefore, our results could not be simply explained by the fact that patients were attending an enjoyable social activity: the training was a complex pathway into an emotional world, leading the patients to regain the ability to manage their emotions much better than patients who were having physiotherapy (i.e., the most common treatment associated with medical therapy). In our opinion, these elements could explain the different outcomes of other types of complementary therapies, for example, dance [13, 14] or martial arts [12].

### 4.3. Limitations of the Study

We are aware of some methodological limitations of our study. First, this is not a randomized study and in Materials and Methods we have explained why we exploited this experimental design. However, we measured several clinical parameters (diagnosis, age, years of disease, onset of disease, education, H&Y, LEDD, and MMSE) and we demonstrated that they were not different across theatre and control groups. In addition, the two groups were scored by blind raters, thus avoiding bias during the evaluations. Obviously, as in similar studies, it was impossible to make patients blind to the treatment condition. Overall, we believe that the limitation of this experimental approach is more theoretical than practical, as it must be supposed that unpredictable and hidden factors systematically affected a certain group.

Second, our sample is relatively small (but similar to those of most studies in this field (e.g., [13–15])). To address this problem, we performed very stringent statistical analyses providing parameters that allow estimation of the effect size and facilitate the interpretation of the substantive as well as the statistical significance of the results [20]. In all instances, we had strong or very strong effect sizes, indicating the consistency of our findings.

Third, we had a relatively high drop-out rate, mainly at the recruitment stage, which was principally due to the length of the treatment. Patients at the beginning of the study were requested to give their availability for 15 months, that is, a very long period of time especially for people affected by a neurodegenerative disorder. Indeed, we are not aware of other studies able to keep patients for such a long period of time.

Fourth, as in the previous study, we included patients with a moderate form of PD because they are likely to fully enjoy the theatrical experience. However, whether this treatment could also be effective on patients with more severe forms of PD, maybe implementing a virtual reality training (e.g., [33]) for those who could not walk, needs to be tested.

Fifth, as clinical scales are self-reported measures, they require the conscious participation of the person, which can alter the final outcomes. Thus, there is a need to collect data such as psychophysiological measurements which can be used to monitor the emotional state of patients independently of their conscious reporting to assess how theatre training changes the processing of affective states of PD patients during the therapy.

## 5. Conclusions

In conclusion, even though this work suffers from some weaknesses and must be viewed as a pilot study, it confirms that theatre represents a valid add-on therapy for PD and it provides some evidence that emotional training improves the patients' mood and thus patients' QoL.

## Supplementary Material


**Movie 1**: This movie shows some example basic exercises performed by some PD patients at the beginning of the training. All patients were ON-therapy.
**Movie 2**: This movie shows some example exercises focusing on the emotional training performed by some PD patients. All patients were ON-therapy.
**Movie 3**: This movie shows an interview with those patients of the theatre group who were willing to speak about their experience at the end of the emotional training. The interviewer asked them the following question: “What did you expected from this training?”
**Movie 4**: This movie shows an interview with those patients of the theatre group who were willing to speak about their experience at the end of the emotional training. The interviewer asked them the following question: “What is your feeling about the results?”

## Figures and Tables

**Table 1:** Exercises included in a typical emotional theatre training session. It must be remarked that some but not all exercises were performed in each session.

Movement training	

*Proprioception*	Visualization; self-awareness; breath exercises; postural exercises

*Basic motor skills practice*	Breathing exercises; spinal column exercises; head exercises; limb exercises; foot exercises; hand exercises; muscle exercises; joint exercises; stretching; strength and flexibility; coordination; dissociation; active/passive motion; balance; memory; sequences; meaningful actions; nonfunctional, expressive movement

*Space*	Body space; personal space; proximal space; medial and distal space; imaging space; spatial cues

*Time*	Internal time; external time; temporal cues; rhythm

*Relationship*	Sound; solo work; couple work; group work; contact

Voice training	

*Proprioception*	Visualization; self-awareness; postural exercises

*Basic motor skills practice*	Spinal column exercises; head/neck exercises; limb exercises; foot exercises; hand exercises; pelvic exercises; shoulder exercises

*Breathing*	Diaphragmatic breathing; movements of abdominal muscles; pelvis exercises; emission of breath for increasing time/with puffs; vocal emission of the consonant “S,” “SH,” and “TS” for increasing time/with puffs

*Resonators*	Vocal emission of the “M” consonant, with closed mouth and rhythmic movements, from low to high pitches

*Vowels emission*	Individual emission of vowels following different orders with a single breath, from low to high pitches, from low to high volume

*Articulation*	Mouth and tongue exercises; facial muscles exercises; articulation of single consonants; exasperated articulations of words; rhythmic articulation of lyrics

*Musicianship*	Performing scales and arpeggios with different syllables/with two-syllable words from low to high pitches in different rhythms, from low to high volume

*Singing*	Improvisation (solo work, couple work, and choral work); learning and performing a song (choral work)

Theatre training	

*Vocal technique*	Breathing; diaphragmatic breathing; vocal exercise; articulation of consonant; syllables and word exercises; increasing and decreasing voice

*Improvisation and experimentation*	Improvisation linked on a given idea; timing and rhythm; variations about the story; including all the partners on stage in a common happening; self-collocate in a drama; fixing the acme; exit the situation; totally enter again in the drama; use of the text; apperceive a personal feeling; communicate it to audience; use of the stage like a chessboard; any step is an emotional stage; Performing with one, two, or three partners; create a story, interpreting and closing it with a comic end. Control and experience the emotional states associated with the story and, finally, reinterpret it with expressing the opposite emotion; exercises of theatrical strategies to be useful for partners; increase and decrease scenic rhythms; deconstruction of a drama, linked to bodily and vocal reps; positive training to prepare to facing audience

*Dramaturgy*	Comedy of Arts techniques; techniques of pantomime; use of the body to create a feature; kind of walking to fix it; study of classic text; dramaturgical analysis; methods to memorize the learned techniques of the Comedy of Arts

**Table 2:** Exercises included in the physiotherapy rehabilitation program. Depending on the single patient's motor and functional status, the physiotherapist could include exercises other than those indicated as basic exercises.

Activities	Basic exercises
*Supine position (15 minutes)*	Diaphragmatic, segmental, and deep breathing exercises; movements to the fullest range of motion of hip, knee, ankle, shoulder, elbow, and wrist; postural changes to lateral and prone position

*Seated position (15 minutes)*	Muscle-stretching of scapular, hip flexor, hamstring, and gastrocnemius; active flexion, extensions, and rotation of upper and lower limbs

*Standing position (15 minutes)*	Standing wall push-up; pelvic mobility (anterior and posterior tilts); side to side tilts; pelvic clock exercise and ball exercise to facilitate sitting control; sit to stand transfer

*Overground gait training (20 minutes)*	Overground gait training (forwards, backwards, and lateral); walking on the spot

*Balance training (15 minutes)*	Weight shifts in both sitting and standing; sitting and standing activities on gymnastic ball

**Table 3:** *p* values obtained from the Shapiro-Wilk test of normality for the two groups of PD patients (theatre group and control group) in all clinical scales and subscales at the beginning (*T*0) and the end (*T*1) of the rehabilitation treatments. *p* values less than 0.05 indicate that the data are not from a normally distributed population and are indicated in bold. UPDRS:Unified Parkinson's Disease Rating Scale; GFQ: Gait and Falls Questionnaire; PDQ39: Parkinson's Disease Quality of Life Scale; PDSS: Parkinson's Disease Sleep Scale.

	Theatre		Controls	
	*T*0	*T*1	*T*0	*T*1
UPDRS I	0.147	0.563	0.109	0.370
UPDRS II	0.18	0.38	0.579	**0.047**
UPDRS III	0.666	0.638	0.596	0.375
UPDRS IV	0.303	0.091	0.175	0.762
Schwab and England Scale	**0.020**	**0.012**	0.051	**0.004**
GFQ	0.148	0.302	0.599	0.684
PDQ39 *(total score)*	0.240	0.542	0.062	0.784
PDQ39 *(mobility)*	0.834	0.526	0.247	0.667
PDQ39 *(activities of daily living)*	0.559	0.703	0.733	0.088
PDQ39 *(Emotional well-being)*	0.303	0.956	0.137	0.825
PDQ39 *(stigma)*	0.133	**0.008**	0.071	0.412
PDQ39 *(social support)*	0.144	0.083	0.333	0.598
PDQ39 *(cognition)*	0.201	0.109	0.143	0.135
PDQ39 *(communication)*	0.071	0.095	0.135	0.174
PDQ39 *(bodily discomfort)*	0.745	0.360	0.916	0.813
Beck Depression Inventory	0.126	0.324	0.103	**0.013**
Apathy Evaluation Scale	0.252	0.291	0.071	0.144
Hamilton Anxiety Rating Scale	0.338	0.286	0.440	0.366
PDSS	0.702	0.341	0.343	0.479
Raven test	0.099	0.051	0.623	0.256
Stroop (word reading time)	0.448	0.659	0.393	0.189
Stroop (color reading time)	0.080	0.262	0.542	**0.025**
Stroop (interference reading time)	0.321	0.080	0.278	0.365
Rey immediate recall	0.778	0.757	0.128	0.088
Rey delayed recall	0.551	0.923	0.086	0.062
Forward digit span	0.197	0.543	0.432	0.354
Backward digit span	0.069	0.123	0.433	0.187
Phonological fluency test	0.382	0.216	0.450	0.416

**Table 4:** Clinical data of PD patients of theatre (TH) and control (CT) groups. For each patient, sex, age, years of education, side of disease onset (L = left; R = right), years since diagnosis, mini-mental state examination (MMSE) scores, Hoehn and Yahr scores (H&Y) in ON and OFF state (see text for definitions), and L-dopa equivalent daily dose (LEDD) (mg) at time *T*0 are given. At the bottom of the table, the *t* values, the corresponding degrees of freedom, the *p* values, and Cohen's *d* values for the comparisons between the two groups are reported. ^*∗*^Equal variances were not assumed. ^§^This patient had a deep brain stimulator.

	Sex		Age		Years of education		Side of onset		Years since diagnosis		MMSE		H&Y		LEDD (mg)	
	TH	CT	TH	CT	TH	CT	TH	CT	TH	CT	TH	CT	TH	CT	TH	CT
1	M	M	72	57	18	13	L	R	17	8	29	29	3	3	1260	936
2	F	F	57	65	13	5	R	R	7	7	29	30	2	3	505	700
3	M	F	66	64	18	13	R	L	7	6	30	30	3	3	1584	500
4	M	F	67	55	18	13	R	L	8	8	29	29	3	3	728	300
5	M	F	61	68	13	18	R	L	12	8	27	28	3	3	480	630
6	F	F	62	57	13	13	R	R	18	10	29	28	3	3	1073	850
7	F	F	60	47	5	8	L	R	11	4	29	27	2	2	739	453
8	F	F	60	50	18	13	R	R	3	9	30	27	1	2	153	500
9^§^	F	M	42	65	12	13	L	L	10	5	25	30	2	3	480	353
10	F	M	56	63	10	13	L	R	4	5	25	29	2	2	327	400
11	M	M	39	72	18	8	R	R	7	18	28	30	3	3	1051	605
12	F	M	64	62	11	13	L	L	8	6	28	30	2	2	200	250

*Mean*			**58.8**	**60.3**	**13.9**	**11.9**			**9.3**	**7.8**	**28.2**	**28.9**	**2.4**	**2.7**	**715**	**539.8**
*SD*			**9.6**	**7.6**	**4.2**	**3.4**			**4.2**	**3.7**	**1.7**	**1.2**	**0.2**	**0.5**	**445.2**	**212.7**

*t-test*			*t*(22) = 0.4		*t*(22) = −1.3				*t*(22) = 0.9		*t*(22) = −1.3		*t*(22) = −1.0		*t*(15.8) = 1.2	
			**p** = 0.67		**p** = 0.21				**p** = 0.38		**p** = 0.22		**p** = 0.31		**p** = 0.24^**∗**^	
			Cohen's		Cohen's				Cohen's		Cohen's		Cohen's		Cohen's	
			**d** = 0.18		**d** = 0.54				**d** = 0.39		**d** = 0.5		**d** = 0.82		**d** = 0.52	

**Table 5:** Mean scores and standard deviations obtained by the two groups of PD patients (theatre group and control group) in all clinical scales and subscales at the beginning (*T*0) and the end (*T*1) of the rehabilitation treatments.

	Theatre		Controls	
	*T*0	*T*1	*T*0	*T*1
UPDRS I	2.9 ± 1.9	3 ± 1.9	2.8 ± 1.7	3.1 ± 1.4
UPDRS II	8.1 ± 4.9	7.8 ± 4.9	6.6 ± 3.8	7.6 ± 4.9
UPDRS III	21.4 ± 9.3	24.2 ± 9.9	21.2 ± 5	22 ± 4.9
UPDRS IV	4.4 ± 3.9	3.2 ± 3.2	3.9 ± 2.2	4.1 ± 1.7
Schwab and England Scale	90 ± 7.4	89.2 ± 6.7	90 ± 9.5	90 ± 10.4
GFQ	20.1 ± 15.8	20.7 ± 15.7	10.5 ± 6.2	10.9 ± 6.4
PDQ39 *(total score)*	58.1 ± 27.1	50.3 ± 22.6	47.3 ± 14	52.2 ± 16.9
PDQ39 *(mobility)*	15.4 ± 9.2	16.8 ± 9.3	12.8 ± 5.7	15.8 ± 5.5
PDQ39 *(activities of daily living)*	9.3 ± 5.3	9.1 ± 4.1	8 ± 2.9	9.7 ± 4.2
PDQ39 *(Emotional well-being)*	11 ± 5.5	8.2 ± 4.9	11.1 ± 3.6	10.9 ± 3.3
PDQ39 *(stigma)*	6.8 ± 3.9	3.5 ± 3.3	3 ± 1.8	3.4 ± 1.7
PDQ39 *(social support)*	2.4 ± 2.1	1.9 ± 1.4	1.9 ± 1.4	1.9 ± 1.1
PDQ39 *(cognition)*	4.6 ± 3.7	3 ± 2.8	2.9 ± 1.5	3 ± 1.5
PDQ39 *(communication)*	2.5 ± 2.3	1.9 ± 1.6	2.1 ± 1.6	2.5 ± 1.3
PDQ39 *(bodily discomfort)*	6.1 ± 2.1	5.9 ± 2.4	5.5 ± 2	5 ± 2.1
Beck Depression Inventory	11.4 ± 5.9	7 ± 5	10.3 ± 5.2	10.4 ± 5.3
Apathy Evaluation Scale	14.9 ± 5.6	10.1 ± 5.3	12.8 ± 6.1	13.7 ± 6.2
Hamilton Anxiety Rating Scale	13.4 ± 5.6	10.2 ± 6.1	13.8 ± 7.2	14.3 ± 6.8
PDSS	20.1 ± 11.1	15.8 ± 9.5	13.9 ± 5.4	14.7 ± 4.3
Raven test	29.1 ± 5.1	29.4 ± 5.5	28.9 ± 3.8	29.2 ± 3.1
Stroop *(word reading time)*	15.8 ± 1.7	13.5 ± 1.9	14.3 ± 1.9	14.8 ± 2.2
Stroop *(color reading time)*	22.3 ± 4.4	21.3 ± 4.6	19.8 ± 3	20 ± 2.7
Stroop *(interference reading time)*	39.5 ± 10.6	38.5 ± 11.8	42.8 ± 8	43.2 ± 6.8
Rey immediate recall	47.8 ± 8.4	48.4 ± 9.3	43.3 ± 9.3	43.9 ± 7.3
Rey delayed recall	9.3 ± 2.2	9.5 ± 2.5	9.3 ± 3	9.8 ± 2.6
Forward digit span	6.8 ± 1.5	6.9 ± 1.6	6.9 ± 1.2	7 ± 1.3
Backward digit span	4.3 ± 1.1	4.2 ± 0.9	4.1 ± 1.2	4.5 ± 1
Phonological fluency test	45.3 ± 15.4	46.1 ± 13.4	38.2 ± 9.8	39 ± 9.1

**Table 6:** Results of the two-way mixed-design ANOVA with GROUP (theatre; controls) as between subjects' factor and TIME (*T*0, *T*1) as within subjects' factor, comparing scores of clinical scales (or subscales, top part of the table) and of neuropsychological tests (lower part of the table). Asterisk (*∗*) indicates subscales of Parkinson's Disease Quality of Life Scale (PDQ39). *η*_*p*_^2^ represents the partial eta-squared; values higher than 0.14 indicate strong effect sizes (see Section 2.5 for further details). *p* values less than 0.05 as well as the corresponding *F* and *η*_*p*_^2^ values are indicated in bold. Unified UPDRS: Parkinson's Disease Rating Scale; GFQ: Gait and Falls Questionnaire; PDSS: Parkinson's Disease Sleep Scale; TR: time for reading words; TC: time for naming color; TI: time for naming color during interference.

	TIME			GROUP			TIME × GROUP		
	*F*(1,22)	*p value*	*η*_*p*_^2^	*F*(1,22)	*p value*	*η*_*p*_^2^	*F*(1,22)	*p value*	*η*_*p*_^2^
UPDRS I	0.83	0.37	0.04	0.004	0.95	<0.001	0.29	0.59	0.01
UPDRS II	0.26	0.62	0.01	0.22	0.64	0.01	1.03	0.32	0.04
UPDRS III	1.55	0.23	0.07	0.19	0.67	0.01	0.44	0.51	0.02
UPDRS IV	1.16	0.29	0.05	0.04	0.85	0.002	1.98	0.17	0.08
GFQ	0.26	0.62	0.01	4.06	0.06	0.16	0.01	0.93	<0.001
PDQ39	0.47	0.49	0.02	0.26	0.61	0.01	3.92	0.06	0.15
Mobility^*∗*^	3.09	0.09	0.12	0.41	0.53	0.02	0.37	0.55	0.02
Activities of daily living^*∗*^	0.57	0.46	0.02	0.06	0.81	0.003	0.86	0.36	0.04
Emotional well-being^*∗*^	**5.4**	**0.03**	**0.19**	0.71	0.41	0.03	**4.2**	**0.05**	**0.16**
Stigma^*∗*^	**14.1**	**0.001**	**0.39**	3.24	0.08	0.13	**23.3**	**<0.001**	**0.51**
Social support^*∗*^	1.44	0.24	0.61	0.12	0.73	0.005	2.82	0.11	0.11
Cognition^*∗*^	**5.13**	**0.03**	**0.19**	0.72	0.41	0.03	**6.3**	**0.02**	**0.22**
Communication^*∗*^	0.14	0.71	0.006	0.01	0.9	0.001	**5.11**	**0.03**	**0.19**
Bodily discomfort^*∗*^	1.26	0.27	0.05	0.82	0.38	0.04	0.31	0.58	0.01
Beck Depression Inventory	**29.6**	**<0.001**	**0.57**	0.27	0.6	0.12	**34.4**	**<0.001**	**0.61**
Apathy Evaluation Scale	**8.78**	**0.007**	**0.28**	0.09	0.76	0.004	**18.93**	**<0.001**	**0.46**
Hamilton Anxiety Rating Scale	1.47	0.24	0.06	0.85	0.37	0.04	2.73	0.11	0.11
PDSS	**4.34**	**0.049**	**0.16**	0.13	0.27	0.06	**8.7**	**0.007**	**0.28**

Raven test	0.65	0.43	0.03	0.13	0.91	0.001	0.13	0.9	0.001
Stroop test (TR)	**6.81**	**0.016**	**0.24**	0.14	0.91	0.001	**14.4**	**0.001**	**0.4**
Stroop test (TC)	0.82	0.37	0.04	1.59	0.22	0.07	2.28	0.14	0.09
Stroop test (TI)	0.06	0.81	0.003	1.15	0.29	0.05	0.35	0.56	0.016
Rey test (immediate recall)	0.24	0.63	0.01	1.84	0.19	0.08	<0.001	1	<0.001
Rey test (delayed recall)	0.94	0.34	0.04	0.04	0.84	0.002	0.1	0.75	0.005
Digit span test (forward)	0.08	0.77	0.004	0.03	0.87	0.001	<0.001	1	<0.001
Digit span test (backward)	0.37	0.55	0.02	0.07	0.8	0.003	0.83	0.37	0.04
Phonological fluency test	0.6	0.44	0.03	2.12	0.16	0.09	<0.001	1	<0.001

**Table 7:** Post hoc analyses of significant interactions obtained in two-way mixed-design ANOVAs [between subjects' factor: GROUP (theatre; controls); within subjects' factor: TIME (*T*0, *T*1), see Table 5]. Data are reported just for those post hoc tests which survived the Bonferroni correction. Asterisk (*∗*) indicates subscales of Parkinson's Disease Quality of Life Scale. *M*_diff_: differences between the means; SD_diff_: standard deviation of *M*_diff_, CI_diff_: confidence interval of *M*_diff_. Other abbreviations are as in Table 6.

	Theatre *T*0 versus theatre *T*1					Theatre *T*0 versus controls *T*0				
	*M*_diff_	SD_diff_	CI_diff_	Cohen's *d*	*p value*	*M*_diff_	SD_diff_	CI_diff_	Cohen's *d*	*p value*
Emotional well-being^*∗*^	2.8	0.9	[0.9, 4.7]	0.72	0.005	—	—	—	—	—
Stigma^*∗*^	3.3	0.5	[2.2, 4.5]	1.2	<0.001	3.8	1.2	[1.3, 6.4]	1.5	0.005
Cognition^*∗*^	1.6	0.5	[0.6, 2.5]	0.63	0.003	—	—	—	—	—
Beck Depression Inventory	4.4	0.5	[3.3, 5.6]	1.07	<0.001	—	—	—	—	—
Apathy Evaluation Scale	4.8	0.9	[2.9, 6.8]	1.18	<0.001	—	—	—	—	—
PDSS	4.3	1.2	[1.8, 6.8]	0.55	0.002	—	—	—	—	—
Stroop test (TR)	2.2	0.5	[1.2, 3.3]	1.71	<0.001	—	—	—	—	—
