# Supplementary material for: Theatre Is a Valid Add-On Therapeutic Intervention for Emotional Rehabilitation of Parkinson's Disease Patients
Source: Parkinsons Dis. 2017 Nov 22;2017:7436725. doi: 10.1155/2017/7436725 (PMC5735633; doi:10.1155/2017/7436725)
Supplement: Supplementary file 1 — Movie 1: This movie shows some example basic exercises performed by some PD patients at the beginning of the training. All patients were ON-therapy. Movie 2: This movie shows some example exercises focusing on the emotional training performed by some PD patients. All patients were ON-therapy. Movie 3: This movie shows an interview with those patients of the theatre group who were willing to speak about their experience at the end of the emotional training. The interviewer asked them the following question: “What did you expected from this training?” Movie 4: This movie shows an interview with those patients of the theatre group who were willing to speak about their experience at the end of the emotional training. The interviewer asked them the following question: “What is your feeling about the results?” [file 7436725.f1.zip › MOVIES CAPTIONS.pdf]

**MOVIES CAPTIONS**

**Movie 1** This movie shows some example basic exercises performed by some PD patients at the beginning of the training. All patients were ON-therapy.

**Movie 2** This movie shows some example exercises focusing on the emotional training performed by some PD patients. All patients were ON-therapy.

**Movie 3** This movie shows an interview with those patients of the theatre group who were willing to speak about their experience at the end of the emotional training. The interviewer asked them the following question: “What did you expected from this training?”

**Movie 4** This movie shows an interview with those patients of the theatre group who were willing to speak about their experience at the end of the emotional training. The interviewer asked them the following question: “What is your feeling about the results?”
